# Omphacinon (unripe olive oil) as a traditional Mediterranean medicinal carrier: an ethnopharmacological appraisal

**DOI:** 10.3389/fphar.2026.1885776

**Published:** 2026-08-05

**Authors:** Elron Zabatani, Zohar Amar, Zipora Tietel, Arnon Dag

**Affiliations:** 1 Department of Land of Israel Studies and Archaeology, Bar-Ilan University, Ramat Gan, Israel; 2 Volcani Institute, Agricultural Research Organization, Gilat Research Center, Rishon LeTsiyon, Israel

**Keywords:** ethnopharmacology, medicinal carrier oil, *Olea europaea*, omphacinon, polyphenols, traditional medicine, unripe olive oil

## Abstract

Omphacinon, the oil from unripe olives (*Olea europaea* L.), occupied a privileged position in Mediterranean materia medica from the Hellenistic period to the late Middle Ages. Sources in Greek, Latin, Aramaic, Hebrew, and Arabic record its use in oral hygiene, dermatological care, antiperspirant preparations, and as a base for medicinal ointments and perfumes; Dioscorides identifies it as the oil best suited to medicinal use. Despite cost and lower yield, ancient practitioners preferred unripe fruit. A preference persisting across more than a millennium and across different medical traditions raises a testable question: do the chemical features of unripe olive oil correspond to the medicinal-preventive advantages attributed to it in the historical record? We work across three bodies of evidence: primary sources (Theophrastus, Dioscorides, Pliny, Columella, the Geoponika, Paulus Aegineta, the Mishnah and Babylonian Talmud, Maimonides, Ibn al-Baytar), archaeological reports from production sites (Pompeii, Herculaneum, Delos, Paestum), and the modern analytical literature on unripe olive oil composition. Botanical names were verified against the Medicinal Plant Names Services (MPNS) and World Flora Online (March 2026); reporting follows the Four Pillars of Best Practice in Ethnopharmacology and the Consensus Statement on Phytochemical Characterisation (ConPhyMP) framework. Modern analyses point to a consistent picture: oil from unripe fruit carries higher levels of polyphenols, tocopherols, and squalene, with lower volatile content and greater oxidative stability than oil from ripe fruit. This profile makes chemical sense of several uses recorded in the historical sources. Antimicrobial activity is relevant to oral hygiene; squalene-mediated emollient effects are relevant to dermatological care; the antioxidant profile favors the oil as a stable carrier for medicated and aromatic preparations. The strength of the evidence varies. Oxidative stability and carrier oil functionality have firm chemical support. Oral health uses are mechanistically plausible and consistent with clinical data on olive oil. Dermatological uses rest on indirect evidence. Antiperspirant claims remain speculative, and the rabbinic claim of depilation is not adequately explained by current chemistry. Omphacinon is best treated as a candidate for evidence-based validation through controlled comparison with mature olive oil. It provides no grounds for therapeutic recommendation.

## 1 Introduction

Omphacinon oil is the term used in antiquity for unripe olive oil. It was employed primarily for medicinal and cosmetic applications, with occasional culinary use among elites. The aim of the present work is to review the medicinal-preventive properties attributed to Omphacinon in historical sources, with particular attention to its use in oral hygiene, dermatological care, and compound medicinal-aromatic preparations, and to evaluate these claims in light of modern analytical and biomedical literature.

The olive tree (*Olea europaea* L.) has long held cultural, historical, religious, and environmental significance throughout the Mediterranean basin. It is generally accepted that olive domestication began in the Fertile Crescent approximately 6,000 years ago (Kaniewski et al., 2012; Zohary et al., 2012), with evidence suggesting that key domestication processes may have occurred in parts of the southern Levant, which subsequently became home to ancient olive cultivation traditions and early olive varieties, with cultivation practices later spreading throughout the Mediterranean world.

Throughout history, olive trees have served multiple essential functions in Mediterranean societies. The fruit was consumed fresh or processed for preservation, while olive oil fulfilled diverse roles: a primary cooking medium, fuel for lighting, a base for medicinal preparations, and a component in various industrial applications (Frankel, 1999). In religious and ceremonial contexts, olive oil held particular importance; biblical sources specify that only the finest grade oil (“shemen zayit zach katit”) was suitable for sacred purposes (Exodus 27:20; Leviticus 24:2), demonstrating the ancient recognition of distinct quality grades for different applications [Pliny (Pliny, 2007) NH 15.5, 15.18].

The ancient perfume and cosmetics industry represents one of the most sophisticated applications of olive oil technology. High-quality olive oil became the cornerstone of luxury cosmetic preparations such as anointing oils, with practitioners developing complex techniques for creating aromatic compounds and therapeutic formulations (Brun, 2000). In later periods, particularly from the medieval era onward (10th century CE and later), olive oil also became central to soap production, with major manufacturing centers producing high-quality products for regional and international trade (Ashtor, 1983; Dag et al., 2020).

Within this context of specialized olive oil applications and quality recognition, Omphacinon oil represents a noteworthy case study for understanding how ancient empirical knowledge may correspond with modern scientific analysis. From an ethnopharmacological perspective, the consistent preference for this oil despite premium cost and lower yield suggests functional advantages that ancient practitioners may have recognized empirically, even without analytical tools to characterize the chemical differences we can now measure.

What is striking, viewed from an ethnopharmacological perspective, is how consistently the unripe fruit was chosen. Greek, Roman, rabbinic, and Arabic-Islamic practitioners all selected it for the most highly valued medicinal-preventive preparations, even though doing so meant earlier harvest, more careful pressing, and substantially lower oil yield. The applications recorded in these sources are wide-ranging. Oral hygiene appears in classical and medieval medical writing. Skin care surfaces in rabbinic, classical, and Arabic sources. Gastrointestinal complaints are mentioned by Dioscorides. The preservation of pharmaceutically active aromatic preparations recurs throughout the recipe literature. A pattern of preference that survives across more than a millennium, in cultural and linguistic contexts as different as Hippocratic Greek medicine and medieval Cairene pharmacy, is unlikely to be arbitrary. It points to functional advantages that practitioners themselves observed in practice, even if they could not name them in chemical terms. Modern analytical work now allows us to ask whether the composition of unripe olive oil supports the uses recorded in those sources. We treat Omphacinon, accordingly, as a candidate for evidence-based validation of traditional practice. Historical claims are compared against current chemical and pharmacological data, and a clear distinction is drawn between uses that find strong chemical support, those that remain at the level of mechanistic plausibility, and those that are still speculative. Several of the recorded applications, in particular oral and dermatological care, have direct counterparts in contemporary preventive medicine; the historical record therefore has more than antiquarian interest.

The evidence assembled here is deliberately cross-cultural. The pharmacological core rests on the Greco-Roman medical writers, chiefly Dioscorides and Pliny, with Theophrastus and Columella for production, while the archaeological evidence for small-scale pressing comes from Italian and Aegean sites such as Pompeii, Herculaneum, Delos, and Paestum as well as from the Levant. The rabbinic corpus contributes a distinct layer of everyday and ritual use, and medieval Arabic sources record the continuity of the same terminology and practice (omphakinon to anfaq). The modern comparative data used to test the historical claims is in part drawn from studies on Levantine cultivars such as Souri, but the chemical pattern at issue, the decline of polyphenols and squalene with ripening, is a general property of Olea europaea rather than a regional one, so the geographic origin of these studies does not narrow the scope of the review. The framing is therefore Mediterranean in reach, with the Levantine sources providing depth of documentation rather than the center of the practice.

## 2 Methodology

This narrative ethnopharmacological review integrates three complementary lines of evidence.

### 2.1 Textual-historical analysis

Primary sources in Greek, Latin, Hebrew, Aramaic, and Arabic were examined for references to Omphacinon/anphakinon and its uses in medicine, cosmetics, and perfumery. Sources consulted include Theophrastus, Dioscorides, Pliny, Columella, the Geoponika, and Paulus Aegineta from the classical tradition, as well as the Mishnah, Tosefta, Babylonian Talmud, Maimonides, and Ibn al-Baytar from the rabbinic and medieval Arabic traditions. Particular attention was paid to descriptions of the oil’s properties, production methods, and preferred applications.

Primary sources were identified on the basis of established familiarity with the classical and rabbinic corpora, building on earlier philological work on ancient perfumery (Zabatani, 2024) and on the anphakinon tradition (Amar, 2026). Three considerations governed inclusion. From the Greco-Roman tradition, the review prioritized the technical and professional literature of the period, that is, the authors who treated agriculture, materia medica, pharmacology, perfumery, and natural history directly. Rabbinic literature is halakhic in purpose, yet it preserves incidental evidence for material and everyday culture; it is most useful here where it corresponds with the classical professional literature and can be read against it. Medieval Arabic sources were included as witnesses to cultural continuity, since they translated and transmitted the Greek terminology into Arabic (omphakinon → anfaq) and document the persistence of the same materials and uses into the medieval period. Sources were set aside when they neither bore on the identity, production, and use of the oil nor added independent evidence beyond later derivative compilation.

### 2.2 Archaeological analysis

Archaeological reports relevant to small-scale olive pressing and perfume production were reviewed to assess whether material evidence supports the literary descriptions. Sites examined include Pompeii, Herculaneum, Delos, Paestum, and various Levantine sites where small pressing installations (Bodedas) have been identified.

### 2.3 Scientific analysis

Modern scientific literature on unripe olive oil composition, oxidative stability, volatile profile, squalene content, polyphenols, and reported bioactivities was surveyed. Searches were conducted in Google Scholar, Web of Science, and PubMed using combinations of terms including ‘unripe olive oil,’ ‘green olive oil,’ ‘olive ripening,’ ‘polyphenols,’ ‘volatile profile,’ ‘squalene,’ ‘oil pulling,’ ‘olive oil skin,’ and ‘olive oil perfumery.’ Additional relevant studies were identified from reference lists of retrieved articles. No date or language restrictions were applied, though the most relevant literature was published in English. Studies were prioritized when they addressed unripe olive oil specifically, reported analytical comparisons across ripening stages, or directly informed historically documented uses; literature on olive oil in general was included only when clearly relevant to mechanistic interpretation. Particular attention was paid to distinguishing evidence specific to unripe olive oil from data concerning olive oil in general, and to differentiating direct clinical evidence from mechanistic plausibility or *in vitro* inference.

Studies were excluded when they concerned olive products unrelated to oil composition or use, or did not inform the historical questions. During revision, the search was extended to terms for individual constituents and quality parameters raised in review, including ‘oleocanthal,’ ‘oleuropein,’ ‘oleacein,’ ‘tyrosol,’ ‘hydroxytyrosol,’ ‘fatty acid composition,’ ‘extraction,’ and ‘composition and maturity.’ Consistent with the design of a narrative review, sources were selected for relevance and authority rather than by exhaustive enumeration. Counts of records at each stage were not tracked, and a quantitative selection diagram is therefore not reported; in a review of this kind, expert selection of the most relevant and authoritative studies, rather than a tally of retrieved records, is the appropriate method.

The combination of these methodologies allows the placement of historical traditions in a scientific context, examining their correspondence with contemporary knowledge while acknowledging the limitations of purely chemical data in validating therapeutic efficacy claims. Scientific plant names were verified against the Medicinal Plant Names Services (MPNS) of the Royal Botanic Gardens, Kew (https://mpns.science.kew.org; MPNS V15, December 2025) and against World Flora Online (www.worldfloraonline.org, accessed March 2026), and nomenclatural authorities were standardized accordingly. The literature search was last updated in March 2026.

### 2.4 Evidence grading and scope of appraisal

Each historically documented use was graded by the strength of its correspondence with modern evidence, on a three-tier scheme reported in Table 1: strong chemical support, mechanistic plausibility, and speculative. The grading distinguishes uses with firm physicochemical backing from those resting on indirect or *in vitro* inference, and separates mechanistic plausibility from clinical demonstration throughout. Formal risk-of-bias and GRADE-type instruments were not applied. These tools are designed for clinical trials and do not transfer to philological or archaeological evidence, nor to the compositional analytical studies that make up the chemical literature reviewed here.

**TABLE 1:** Critical appraisal of traditional claims for Omphacinon oil against modern evidence.

Traditional claim	Primary source(s)	Modern evidence type	Strength of support	Main limitation
Perfume carrier oil/oxidative stability	Pliny, Theophrastus, Dioscorides, recipe literature	Analytical chemistry, experimental reconstruction	Strong	Effects are formulation-specific
Oral hygiene (oil rinsing)	Dioscorides (1949) I.29, Pliny (2007) XXIII.79	Meta-analyses on olive oil pulling	Moderate	No trials on unripe olive oil specifically
Dermatological use (skin refinement)	Babylonian Talmud, Megillah 13a; Pesachim 43a	Squalene and emollient literature	Weak–moderate	No clinical data specific to unripe oil
Antiperspirant effect	Dioscorides I.29	Antibacterial and tannin literature	Weak/speculative	Mechanism not demonstrated for olive oil

Reporting follows the Four Pillars of Best Practice in Ethnopharmacology (Heinrich et al., 2022) and the ConPhyMP framework, in keeping with the standards set by Frontiers in Pharmacology for the Ethnopharmacology section. As this is a historical-critical review and not a study of newly prepared plant material, items in the ConPhyMP checklist that pertain to fresh extracts (voucher specimens, batch numbers, certificates of analysis, originally generated chromatographic data) are not applicable; reviewed analytical literature is cited in the relevant places, and methods of authentication, quantification, and reference comparison are those reported in the cited studies. Completed ConPhyMP Supplementary Tables S1 and S2, with section-by-section cross-references to the manuscript, are provided as Supplementary Material. AI-assisted language editing was used during manuscript preparation; the tool, scope of use, and verification procedure are described in the Acknowledgements.

## 3 Omphacinon oil in antiquity

### 3.1 Description of the oil and its uses

Unripe olive oil is mentioned in classical sources as Omphacinum (ὀμφάκινος) and in rabbinic literature as “anphakinon” (אנפקינון). The name is derived from the Greek word *omphax* (ὄμφαξ), meaning unripe fruit.

Theophrastus (371–287 BCE) notes that this oil should be used when it is fresh, because old oil, more than a year after its production, has become viscous and useless [Theophrastus (2000), Concerning Odours 15]. The observation indicates ancient awareness of oil degradation processes and the importance of freshness for maintaining desired properties.

Dioscorides (first century CE) indicates that the oil was considered the best for medicinal use and served as a base for ointments. In external treatment, its application inhibits the secretion of sweat from the body; in internal treatment, it was also considered beneficial for abdominal pain and intestinal problems (Dioscorides I.29). Dioscorides and Pliny note that rinsing the mouth with the oil was beneficial for teeth and gums hygiene [Dioscorides (Dioscorides, 1949) I.29; Pliny (Pliny, 2007) XXIII.79].

The high price of this oil led to its use mainly by the upper class. Beyond medicinal applications, it had limited culinary use among elites, but its primary application appears to have been cosmetic, being considered an oil beneficial for hair removal and skin refinement (Babylonian Talmud, Menachot 86a; Pesachim 43a; Megillah 13a; Moed Katan 9b). Read alongside the medical record from Dioscorides and Pliny, the rabbinic descriptions of skin refinement may reflect the same dermatological-preventive logic as the oral applications, a logic that would rest on the higher polyphenol and squalene content of the unripe fruit and the antimicrobial and emollient activities reported for these compounds. Despite the halo surrounding its properties, its use in worship at the Temple in Jerusalem was controversial as it was considered an “incomplete” oil (Mishnah, Menachot 8:3; Tosefta, Menachot 8:9; Babylonian Talmud, Menachot 86a).

In medieval literature this oil was called “anfaq” (انفاق) (Ibn al-Baytar, 1874), but many began to use the name for high-quality mature olive oil, one of the export products of the Land of Israel in the Middle Ages (Amar, 2000), while unripe olive oil was called “zeit al-falastin” (Renaud and Colin, 1934). The oil is described as being pressed from green olives with a bitter taste [Maimonides, 1992, Commentary on the Mishnah, Menachot 8:3].

### 3.2 Method of oil production

#### 3.2.1 Harvest time

Classical sources indicate that the harvest time for Omphacinon oil occurred when the fruits are large, green, have not yet changed their hue, and at a stage where the beginning of ripening signs can be seen [(Columella, 1954) XII.54; Geoponika (Dalby, 2011) IX.19].

In the Talmud, Omphacinon oil is described as “oil that has not brought a third.” The amount of oil in green, unripe olives is lower by half or a third compared to ripe olives, with oil constituting 10%–15% in green fruit compared to 20%–30% in ripe fruit. The definition by the Sages that it is oil “that has not brought a third” therefore refers to the stage where one-third of the final oil yield can be obtained from the fruit (Amar, 2026). Based on recent studies, the harvest was ca. 3 months earlier than the common timing for oil production (Dag et al., 2011).

#### 3.2.2 Oil production based on literary sources

Preference was given to harvesting a small quantity, manually and meticulously, so that the olives would not fall to the ground and be damaged. After harvesting, the olives were cleaned and placed under a press or weight, and gentle, non-aggressive pressing was performed. The oil produced had to be collected carefully using a ladle and transferred to a vessel. The oil was stored in a cool, dry place (Columella XII.54; Geoponika IX.19).

These descriptions suggest that the pits were not crushed and that oil production was solely from the olive flesh. At this stage the olives do not have a particularly high percentage of liquids, which led to the fact that the extraction of oil was more difficult than from ripe olives and was not suitable for production in a large oil press, but rather for a smaller pressing facility, a local Bodeda suitable for producing a high-quality liquid. Only a limited number of practitioners appear to have been engaged in producing this oil.

#### 3.2.3 Oil production based on archaeological evidence

**FIGURE 1:**
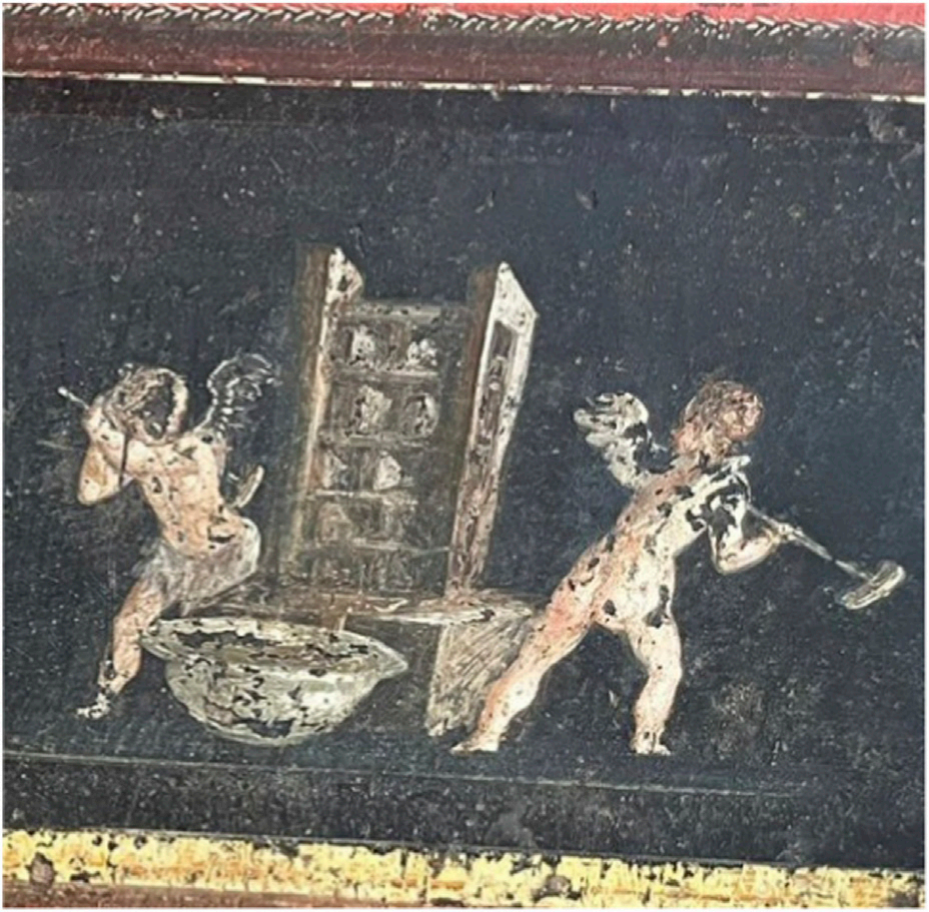
Wall painting from the House of the Vettii, Pompeii (first century CE), depicting a small dedicated oil press (wedge press) used for producing high-quality olive oil for perfume preparation. Photo by David Goldberg, 2023; reproduced with permission.

In Rome, excavations at Pompeii and Herculaneum from the first century CE uncovered several wall paintings depicting a small, dedicated oil press. These presses are thought to have been used for producing high-quality olive oil for perfume preparation. The installation was a type of “Bodeda,” a square or round stone slab with a carved circle connected to a sloping channel leading to a vessel that collected the liquids. Olives were placed on the surface, probably in a woven basket, between two vertical beams with a crossbeam. Within this frame, wooden wedges were forcibly inserted using blows from small hammers (wedge presses). The oil flowed into the peripheral groove and from there through the channel was collected into a bowl (Figure 1).

In Delos and Paestum, several oil presses and Bodedas of this kind were found inside perfume shops, where customers could observe the production process as depicted in the wall painting from Pompeii (Brun, 2000; Mattingly, 1990).

In the Land of Israel area many Bodedas were found, but the connection between them and the production of Omphacinon oil is not certain, and most of them likely served for small-scale production of high-quality olive oil. Eitam suggests that some were intended for base oil for perfumes, based on findings such as crushing and grinding tools and perfume vessels at sites like Tel Gath, even without typical pressing facilities (Eitam, 2022).

### 3.3 Omphacinon oil in the perfume industry

#### 3.3.1 Oils in the perfume industry

Although the historical record on Omphacinon places considerable weight on its use in compound perfumes, this material was rarely a luxury item in the modern decorative sense. Many of these preparations were applied directly to skin and to the oropharyngeal region, and several were marketed and used as medicated unguents whose carrier oil had a recognized hygienic function. Reading the perfumery sources alongside the medical sources is therefore not a digression: in antiquity, the categories overlap. The selection of a polyphenol-rich, oxidatively stable carrier oil shaped both the aromatic performance and the therapeutic-preventive profile of the finished preparation, and the present section examines that selection on both counts.

The perfume production industry underwent change and became more sophisticated over the years. A particularly effective method for producing perfumes is distillation, which is still used today. Most researchers agree that distillation technology was not known in the West during the classical period (Forbes, 1948), and although the principles of distillation were formulated during this period, distillation in stills began only with the appearance of Muslim chemists in the ninth–tenth centuries CE (Hassan and Hill, 1986).

Perfume production in the classical period relied on vegetable oils, into which the scents from aromatic raw materials were imparted using various methods. Although many vegetable oils were used, only some were considered suitable for use as oils for the perfume industry (Table 2) (Forbes, 1955). Selection of the carrier oil mattered for medicinal-preventive reasons as well as for fragrance: a base oil with antimicrobial polyphenol activity contributes to the stability and hygienic profile of the finished preparation, particularly when the perfume is applied directly to skin or used in oral or oropharyngeal contexts.

**TABLE 2:** Oils used as a base for perfume in the classical period.

Oil name	Scientific name
Mature olive	*Olea europaea* L.
Unripe olive (Omphacinon)	*Olea europaea* L.
Washed (emulsion)	*Olea europaea* L.
Sesame	*Sesamum indicum* L.
Walnut	*Juglans regia* L.
Bitter almonds	*Prunus dulcis* (Mill.) D.A.Webb
Moringa	*Moringa peregrina* (Forssk.) Fiori
Egyptian balanites	*Balanites aegyptiaca* (L.) Delile

The selection of oil from this limited list appears deliberate rather than arbitrary, suggesting that ancient practitioners recognized distinct properties in these oils. Matching of oil to spices was done carefully and was an integral part of the perfume production method. Such matching appears to have followed the shared properties of the base oil and the perfume ingredients whose scent was imparted to it.

A deliberate match was made between the characteristic scent of the oil and that of the perfume ingredient, so, for example, the delicate scent of sesame oil connected as a base oil to the delicate scent of roses (Theophrastus, Concerning Odours IV.20).

Contrary to the common explanation today, that a neutral scent is the important measure in matching oil for use as a base for perfumes, it appears from the sources in classical literature that the ancients attributed great importance to the property of astringency in oils in the perfume industry, to the extent that they called the base oils for perfumes “astringents” (Pliny XIII.7).

Our hypothesis is that their perception was that the “penetrating” scent of the oil also helped to intensify the scents imparted to the oil and consequently the scent of the perfume. The same perception was also prevalent regarding the burning of incense, like the description in rabbinic literature about the role of galbanum (*Ferula gummosa* Boiss., syn. *F. galbaniflua* Boiss. & Buhse) in enhancing and sharpening the other scents of incense ingredients that were burnt with it in the Temple in Jerusalem (Babylonian Talmud, Kritot 6b).

#### 3.3.2 The use of omphacinon oil in the perfume industry

Analysis of 28 recipes for popular perfumes in the Roman period shows that the vast majority contain olive oil as the carrier base for aromatic components. Pliny usually mentions Omphacinon, while Dioscorides brings it in several recipes, and in others, olive oil is mentioned generally, which includes Omphacinon (Forbes, 1955).

Omphacinon oil has a concentrated herbaceous scent and possesses astringent properties, and it was therefore matched in several recipes to perfume plants with a heavy or strong scent. Among these recipes are perfumes produced from plants such as mastic (*Pistacia lentiscus* L.) (Paulus Aegineta (Paulus, 2000) VII.20), myrtle (*Myrtus communis* L.) (Dioscorides I.48), galbanum (*Ferula gummosa* Boiss.) (Dioscorides I.71; Pliny XIII.9; Paulus VII.20), henna (*Lawsonia inermis* L.) (Dioscorides I.65; Pliny XII.109; XIII.13), saffron (*Crocus sativus* L.) (Dioscorides I.62; Paulus VII.20), and nard (*Nardostachys jatamansi* (D.Don) DC.) (Dioscorides I.75; Pliny XIII.15).

In the Talmud there is a controversy regarding the identification of the oil of myrrh in which Queen Esther bathed for 6 months (Esther 2:12). One opinion, attributed to Rav Yehuda, is that it is ‘anphakinon’ oil, with the reason being functional because it removes hair and refines the flesh (Babylonian Talmud, Pesachim 43a; Megillah 13a; Menachot 86a). The functional rationale offered by the rabbinic source includes both depilation and skin refinement. Of the two, only the skin-refinement claim maps onto a recognizable medicinal-preventive profile, since the higher polyphenol and squalene content of the unripe oil offers a chemical basis for emollient and antimicrobial effects on the skin. Depilation, by contrast, is not adequately explained by current knowledge of olive oil chemistry; it remains historically significant but pharmacologically open, and is best treated here as a research gap rather than as a chemically substantiated effect.

Previous experimental reconstruction of ancient perfume recipes demonstrated that the effect of Omphacinon oil on the scent of the perfume varies across compositions. In recipes describing the production of perfume from saffron and from nard, two possible oils appear: Egyptian balanites oil and Omphacinon oil. Practical testing revealed that with saffron, the perfume produced with Omphacinon oil was more enhanced, while in the case of nard perfume the result was the opposite (Zabatani, 2024).

Earlier experimental work on reconstructing anointing oil production following post-biblical sources (Exodus 30:23-25) (Amar and Zabatani, 2025) examined whether changing from mature olive oil to Omphacinon oil would contribute to the oil’s scent. Results indicated that there was no pronounced difference in scent. It is possible that despite the scent not being enhanced, Omphacinon oil was used solely because of its long shelf life, which suited the functional role of anointing oil that was used for an extended period.

The use of Omphacinon in ancient perfumery may thus have served a dual functional purpose: in some formulations, potentially enhancing the aromatic characteristics of specific fragrance materials through chemical interactions; in others, extending product stability and shelf life through its elevated antioxidant content. Beyond fragrance, the antimicrobial and antioxidant activities of the polyphenol fraction give the carrier oil itself a hygienic dimension that is particularly relevant when the finished preparation is applied to skin or mucous membranes. The consistent preference for this oil despite its premium cost and lower yield suggests that ancient practitioners perceived functional advantages sufficient to justify its use in high-value preparations. The repeated preference suggests that ancient perfumers recognized practical advantages, even if they lacked a chemical vocabulary for describing them.

## 4 Modern research

### 4.1 Basic chemical characteristics of virgin olive oil and their bioactivities

Among natural emollients, olive oil has been distinguished for skin treatment for millennia. The oil has a high percentage of oleic acid, which together with the essential linoleic and linolenic fatty acids also present in olive oil plays an important role in the barrier function of the skin, facilitating the entry of active agents to its deep layers, reducing transepidermal water loss, and improving environmental protection (Brenner, 2006; Čižinauskas et al., 2017; Dayan, 2005).

Virgin olive oil is also rich in phenolic compounds (tyrosol, hydroxytyrosol, caffeic acid, oleuropein, and others) with essential antioxidant action (Djalil et al., 2019; Ichihashi et al., 2018; Viola and Viola, 2009). Antioxidants are considered anti-aging agents for skin care (Djalil et al., 2019; Ichihashi et al., 2018). The application of virgin olive oil on human skin can also contribute to improving various skin conditions, such as atopic dermatitis, psoriasis, and eczema, by maintaining moisturization of dry and flaking skin and thus making it smoother and more pliable (Brenner, 2006; Viola and Viola, 2009).

### 4.2 The effect of the ripening process on oil composition

In general, virgin olive oil extracted from green-unripe fruit is characterized by a high level of polyphenols and high stability (Dag et al., 2011; Stefanoudaki et al., 2009; Garcia et al., 1996), thereby enhancing antioxidant activity, oxidative stability, and shelf life. Bioactivities attributed to these compounds may also be enhanced when their levels are high.

Maturation of the olive fruit is a long, slow process that lasts several months (Lechhab et al., 2022). During this time, oil content in the fruit increases with maturation. At the same time, levels of free fatty acids in the oil rise as the ripening process progresses, with a simultaneous reduction in polyphenols, tocopherols, chlorophyll, carotenoids, and oxidative stability, as well as in the oleic:linoleic ratio and moisture content (Al-Maaitah et al., 2009).

Table 3 summarizes the relative differences in key chemical constituents between unripe and mature olive oil, based on the studies reviewed.

**TABLE 3:** Comparative chemical profile of unripe (Omphacinon) versus mature virgin olive oil and functional implications.

Compound category	Relative level in unripe oil	Functional implication	Key references
Total polyphenols	Higher	Higher oxidative stability; antioxidant activity	Dag et al. (2011), Garcia et al. (1996)
Tocopherols	Higher	Antioxidant protection	Al-Maaitah et al. (2009)
Squalene	Higher	Skin emollient properties	Nsir et al. (2017), Huang et al. (2009)
Total volatiles	Lower	Suitable as neutral carrier oil base	Psathas et al. (2022)
C6 volatiles (lipoxygenase pathway)	Lower	Reduced aroma interference from carrier	Karagoz et al. (2017)
Free fatty acids	Lower	Better oil quality and stability	Al-Maaitah et al. (2009)
Oxidative stability	Higher	Extended shelf life of oil and perfume formulations	Dag et al. (2011), Stefanoudaki et al. (2009)
Oil yield	Lower (10%–15% vs. 20%–30%)	Higher production cost; limited availability	Dag et al. (2011)

### 4.3 Volatile profile and aromatic properties

The volatile profile of the olive also changes significantly with maturation, with low alcohol and high ketone levels in the unripe oil, increasing and decreasing, respectively, as the process progresses. Total volatile content is also low in the unripe oil (Psathas et al., 2022), increasing to a certain point and then decreasing. C6 volatiles, a group of compounds derived from the lipoxygenase pathway and significant to olive oil aroma, are also low in the unripe oil, in addition to esters (Karagoz et al., 2017).

The accepted method for sensory evaluation of olive oil aroma is by a panel of trained tasters certified by the International Olive Council. The positive aromas of olive oil are defined under the general criterion of fruitiness on a scale of 1–10 according to the intensity of the aroma. Fruitiness is generally divided into ‘ripe fruitiness’ characteristic of ripe olives and ‘green fruitiness’ characteristic of green olives, typically fruity with reminiscent of freshly cut grass aromas. The source of ‘green’ aromas is in C5-C6 aldehydes, alcohols and esters, which are significantly more abundant in the unripe fruit (i.e., low maturity index), e.g., *Z-*3-hexenol, *Z*-3-pentenol, Z-3-hexenal, *E*-2-hexenal, hexanal, hexyl acetate (fruity, apple-pear aroma), methyl acetate and *Z*-3-hexenyl acetate (fresh green fruit) (Morales et al., 2013).

### 4.4 Squalene and other specialized metabolites

Squalene, a triterpene present in the oil, is the main component of skin surface polyunsaturated lipids and shows advantages for the skin as an emollient and antioxidant, and for hydration and antitumor activity (Huang et al., 2009). Squalene levels were reported to be high in unripe oil and decrease with maturation (Nsir et al., 2017), which may help account for the skin benefits attributed to unripe olive oil in the historical sources.

Changes in chemical composition along the olive fruit ripening may help explain the uses described for unripe olive oil. The high content of antioxidants (polyphenols, tocopherols, and squalene), which also provides high oxidative stability, may give stability to the perfume when used as a base oil, stabilize the scent by preventing oxidation of scent compounds in the perfume itself, and provide a long shelf life. Squalene may explain the use for softening the skin. The low level of volatiles is also suitable from this perspective for the perception that the scent of the base oil should be relatively weak, to allow the perfume scent to be expressed.

The presence of phenolic alcohols, mainly tyrosol and hydroxytyrosol, alongside their secoiridoid derivatives, mainly oleocanthal, oleuropein, and oleacein and their aglycones, as well as other key olive polyphenolic compounds significantly contribute to the biological activity, imparting anti-inflammatory effects (Warias and Kurkiewicz-Piotrowska, 2025), alongside antioxidant and immunomodulatory bioactivities (Nunes et al., 2022). Specifically, oleocanthal and oleuropein were reported to possess hydration, elasticity, wrinkle reduction, and photoprotection properties, playing major roles in skin aging (Warias and Kurkiewicz-Piotrowska, 2025). Hydroxytyrosol, tyrosol, and oleocanthal have also been shown to enhance wound healing through action on fibroblasts related to tissue regeneration (González-Acedo et al., 2023). Other skin effects of olive polyphenols include induction of cell proliferation in keratinocytes, increasing the expression of CDK2 and CDK6, acting as immunomodulators, also improving the cell migration through the activation of some tissue remodeling factors (Melguizo-Rodríguez et al., 2022). Moreover, other olive oil compounds, e.g., triterpenes, were also shown to possess desirable effects on the skin, demonstrating skin antiaging, photoprotective, biostimulant and regenerative, and anticarcinogenic properties (Melguizo-Rodríguez et al., 2022).

The compounds described in olive oil, especially polyphenols, show antibacterial activity against skin pathogens *in vitro* (Nunes et al., 2022; Visioli et al., 1998). General olive oil literature provides only indirect support for the historical claim that the oil prevents perspiration: antibacterial effects may be relevant to odor control, but they do not in themselves demonstrate a true antiperspirant effect. In addition, tannins, a specific group of polyphenols also present in olive oil (Malenčić et al., 2008), have been reported to act as antiperspirants, forming a gel-like plug, using the same mechanism as commercial antiperspirants (Teerasumran et al., 2023), though this mechanism has not been specifically demonstrated for olive oil application.

Oil pulling using olive oil was found in a meta-analysis to reduce the number of bacterial colonies in saliva (Peng et al., 2022), and another review article found that it improves several measures of oral hygiene (Gbinigie et al., 2016). These findings are broadly compatible with historical reports of oral use by Dioscorides and Pliny, though they do not establish a specific advantage for unripe olive oil.

### 4.5 Chemical interactions between base olive oil and scent materials

Research has indicated that the carrier medium influences scent perception. It can be hypothesized that the matching between the base oil and the perfume is based, among other things, on the formation of chemical bonds between them. These bonds may improve the solubility of the perfume in the base oil and thus affect the perception of the compounds in the perfume, or the extent of their release from the matrix, allowing for delayed release. It has been suggested that polymers, including polyphenols, may play a role in the delayed release of volatile compounds (Wei et al., 2020).

### 4.6 Carrier oil functionality: Physicochemical basis for perfume applications

Several physicochemical properties distinguish unripe olive oil from the mature form, and they help explain its particular suitability as a perfume carrier. Volatile content is one of them. Oil from unripe fruit is itself low in C6 aldehydes and related compounds, and so it interferes far less with the volatile profile of the fragrance materials added to it. The intended aroma of the finished preparation comes from the chosen ingredients rather than from the carrier. This matters most in compound perfumes, where authenticity and clarity of the dominant note carry both commercial and ritual significance.

The elevated polyphenol fraction is likely to contribute to performance through more than one route. Antioxidant protection is the most direct of these. The polyphenols protect both the lipid matrix of the carrier and the volatile fragrance compounds dissolved within it, and so extend the useful life of the preparation. Beyond protection, the polyphenolic matrix may also alter how those volatiles leave the oil. Weak chemical interactions between polyphenols and aromatic molecules can plausibly affect solubility and rate of evaporation, contributing to a slower, more gradual release of scent (Wei et al., 2020). When the carrier oil is itself rich in antimicrobially active phenolics, the medicinal-preventive value of the finished preparation extends past its aromatic role: skin contact would deliver a low but continuous polyphenol exposure, with possible benefits for skin microbiota balance.

These physicochemical properties may help explain why ancient perfumers selected Omphacinon oil despite its higher cost and lower production yield. The functional advantages in formulation stability, scent preservation, and potentially enhanced aromatic performance could justify the premium investment, particularly for high-value perfume products intended for extended storage or use by elite clientele. In practical terms, this amounts to deliberate material choice based on observed performance, rather than accidental preference, even in the absence of modern chemical understanding of the underlying mechanisms.

### 4.7 The effect of cultivation and production methods on oil quality

Scientific research examining various factors affecting olive oil and its composition showed that in manual harvesting, the level of polyphenols in the oil is higher than in mechanical harvesting. In addition, in the traditional “Souri” variety of the Land of Israel grown under rain-fed conditions (compared to the modern “Barnea” variety that is irrigated), the level of polyphenols and the level of pungency and bitterness decrease with the increase in irrigation doses, and the highest polyphenol content was measured in trees that are not irrigated or at the lowest irrigation levels (Bashir, 2009; Dag et al., 2014).

These cultivation and processing factors may help explain the quality differences that made traditionally produced Omphacinon oil particularly valued. Manual harvesting and minimal irrigation, practices described in ancient sources, would likely have preserved higher polyphenol concentrations, contributing to the oil’s stability and organoleptic properties. The careful, gentle pressing methods documented in classical texts (Columella XII.54) similarly would have minimized degradation and oxidation during extraction.

Beyond the different aroma between oil produced from unripe olives and oil produced from ripe olives, oil produced from unripe olives has high values of polyphenols and other antioxidants, which give it high stability against oxidation and a longer shelf life. When used as a base oil in perfumery, these antioxidants also protect the perfume compounds from chemical degradation through oxidation, thereby extending the stability and shelf life of the entire perfume formulation.

### 4.8 Current uses of unripe olive oil

Today, olive oil from unripe olives is mainly produced from specific varieties, for example, the Picual variety (Gómez-Rico et al., 2008), Cornicabra, Agoureleo Chalkidikis, or Chondrolia Chalkidikis, and is used for culinary purposes, as it is considered to have a particularly high-quality taste and health-promoting properties.

## 5 Discussion: ethnopharmacological appraisal of omphacinon as a medicinal carrier oil

A caution precedes the appraisal that follows. The practitioners who chose unripe olive oil did not work with the concepts used here to explain their choices. They had no access to chromatography, no notion of polyphenols or oxidative stability, and no model of skin microbiota. Their knowledge was empirical and sensory, built from taste, smell, touch, and the observation of how a preparation behaved over time, and it was organized within the humoral framework that governed Greco-Roman and later Arabic medicine, in which substances acted through qualities such as heat, cold, dryness, and astringency rather than through the mechanisms described below (Nutton, 2024; Pormann and Savage-Smith, 2007). Reading modern chemistry into these sources therefore carries a risk of anachronism. The argument made here is not that ancient practitioners understood the chemistry of the oil, but that the practices they arrived at empirically can, in some cases, be accounted for in modern chemical terms. The correspondence runs in one direction. Chemical analysis may explain why an empirically selected practice was effective; it does not show that the ancient explanation and the modern one describe the same thing. Where the two systems share a word, such as the “astringency” attributed to perfume base oils (Section 5.3.4), the term belongs to the ancient sensory and humoral vocabulary and does not map cleanly onto its modern chemical sense. Holding this distinction in view keeps the ethnopharmacological reading honest: it treats the historical record as a guide to practices worth testing, not as a source of validated mechanisms (Heinrich et al., 2022).

### 5.1 Chemical composition and traditional uses of omphacinon oil

The fact that Omphacinon oil extraction resulted in less than 50% oil yield in comparison to virgin olive oil extracted from ripe fruit indicates specific characteristics of this oil when used for perfume production. Modern scientific findings provide the strongest support for the physicochemical rationale behind Omphacinon’s use in perfumery, particularly with respect to oxidative stability, shelf life, and carrier oil performance. By contrast, support for medicinal and cosmetic uses remains uneven and is often indirect rather than clinically demonstrative. The chemical analysis of olive oil produced from green olives indicates that it is an oil particularly rich in phenolic compounds, tocopherols, and squalene, which give it improved oxidative stability compared to oil produced from ripe olives (Dag et al., 2011). This stability corresponds with the historical descriptions of Omphacinon oil as a quality raw material in the ancient perfume industry.

The chemical profile of Omphacinon, as it can be inferred from analyses of early-harvest olive oil, is distinguished by four main categories of constituents. First, the triacylglycerol fraction is dominated by oleic acid (C18:1, typically 60%–80% of FAME), which confers low susceptibility to oxidative rancidity. Second, phenolic compounds, principally oleocanthal, oleuropein, oleacein, hydroxytyrosol, and tyrosol, are present at concentrations significantly higher than in ripe-fruit oil; these are responsible for the characteristic pungency and bitterness of fresh unripe oil and underlie its antioxidant, anti-inflammatory, and antimicrobial bioactivities. Third, tocopherols (primarily α-tocopherol) and squalene contribute further antioxidant capacity and emollient properties valued in topical and cosmetic use. Fourth, the volatile profile at early harvest is low in total content but enriched in C6 compounds derived from the lipoxygenase pathway, giving the oil its distinctive green, herbaceous character without the overpowering fruitiness of ripe oil, a property consistent with its use as a perfume base. Together these features situate Omphacinon chemically as an oil of high bioactive density and low tendency toward sensory interference, properties that cohere with its historical uses as both a medicinal carrier and a perfumery base.

As for quantification of compounds in Omphacinon, i.e., in olive oil produced early from fruit with low maturation index, literature results in ‘Souri’ show that total polyphenol levels are 150–300 mg/kg (Dag et al., 2011). Nevertheless, the reported levels of total polar phenolic compounds are within a wide range of values, from 50 to 1,000 mg/kg, usually ranging between 100 and 300 mg/kg (Kalogeropoulos and Kaliora, 2015). Although variance has been reported between Turkish (oleuropein at 21 mg/kg), Tunisian and Algerian olives (>1,000 mg/kg and 126 mg/kg, respectively; hydroxytyrosol at 232 mg/kg, tyrosol at 37 mg/kg, oleuropein aglycone at 737 mg/kg), and Greek varieties (500–1,000 mg/kg), they all follow the same trend of high polyphenolic content at early stages, decreasing with maturation.

Fatty acids are the main constituents of olive oil, playing a crucial role in improving skin texture, enhancing elasticity, and promoting softness (Warias and Kurkiewicz-Piotrowska, 2025). The unsaturated fatty acids in olive oil help strengthen skin barrier function and improve smoothness and elasticity. Restoration of skin barrier can be facilitated by unsaturated fatty acids. Additionally, olive oil’s consistency allows it to form an occlusive barrier on the skin surface, hence enabling skin hydration. Oil’s effectiveness in preventing and alleviating the appearance of stretch marks is attributed to the presence of oleic acid and unsaturated fatty acids, in addition to squalene and polyphenols (Warias and Kurkiewicz-Piotrowska, 2025). Representative data shows that olive fatty acid profile comprises 7.5%–20% palmitic acid (C16:0), 0.3%–3.5% palmitoleic acid (C16:1), 0.5%–5% stearic acid (C18:0), 55%–83% oleic acid (C18:1), 3.5%–21% linoleic acid (C18:2) and ≤1% linolenic acid (C18:3) (Ouni et al., 2016; Warias and Kurkiewicz-Piotrowska, 2025). However, as olive oil types vary in their fatty acid composition, this may influence their dermatological properties. Results regarding the modifications in olive oil’s fatty acid composition with maturation on the range of fatty acid contents are mixed: while some reports presented an increase in C16:0 and C16:1 with ripening, alongside a decrease in C18:0 and C18:1 (Ouni et al., 2016), others reported a decrease in C16:0 and C16:1, with an increase in C18:0 and C18:1 (Amanpour et al., 2019), although both authors agreed on an increase in C18:2. Other works reported no significant changes in fatty acid composition with maturation (Greco et al., 2022).

The preference mentioned in historical sources by Theophrastus for fresh Omphacinon oil over aged oil is supported by modern research, which shows that despite the improved stability of unripe virgin olive oil compared to regular virgin oil, it also undergoes oxidation and aging processes over time. The high antioxidant content may, however, help explain the preference given by the ancients to this oil in the production of perfumes intended for prolonged use.

### 5.2 Interactions between base oil and fragrance materials

Regarding the role of olive oil as a base oil, studies have shown that the medium influences fragrance perception. The matching between the base oil and the perfume, as reflected in ancient recipes, may be based on the formation of chemical bonds between them. These bonds affect the solubility of the perfume in the oil, the perception of the compounds, and the rate of their release. The high concentration of polyphenols in Omphacinon oil may bind with volatile compounds, potentially slowing their evaporation and extending the perfume’s scent duration.

There is some support for the hypothesis that polyphenol-rich matrices modulate volatile release, particularly from polymer chemistry research (Wei et al., 2020), though direct experimental validation specific to olive oil polyphenols and perfume compounds would be needed to confirm this mechanism. Variable results observed in experimental reconstructions, with Omphacinon enhancing saffron perfumes but not nard perfumes (Zabatani, 2024), suggest that interactions are compound-specific rather than universal, indicating a more complex relationship than simple oxidative protection alone.

### 5.3 Medicinal and cosmetic aspects: evidence quality and limitations

The descriptions in historical sources regarding the use of Omphacinon oil for medicinal and cosmetic purposes show varying degrees of correspondence with modern scientific research. A critical appraisal reveals important distinctions between chemical plausibility and clinical validation.

#### 5.3.1 Oral health applications

Historical claim: Dioscorides and Pliny in the first century CE described the oil as beneficial for teeth and gums hygiene through oil rinsing.

Modern evidence: Meta-analyses indicate that oil pulling with olive oil reduces bacterial colonies in saliva (Peng et al., 2022) and improves measures of oral hygiene (Gbinigie et al., 2016). These studies, however, examined general olive oil, not specifically unripe olive oil.

Limitations and gaps: No clinical trials have compared unripe versus mature olive oil for oral health outcomes. While the higher polyphenol content of unripe oil suggests potentially enhanced antimicrobial activity based on *in vitro* studies (Visioli et al., 1998), this theoretical advantage has not been demonstrated clinically.

Future research needed: Controlled clinical trials comparing unripe and mature olive oil for oil pulling, with microbiological and clinical periodontal outcomes, would be required to validate any specific advantages of Omphacinon oil for this application.

#### 5.3.2 Dermatological uses

Historical claim: Traditional use for “refining the flesh” and skin conditioning.

Modern evidence: Squalene demonstrates emollient properties (Huang et al., 2009), and olive oil fatty acids support skin barrier function (Brenner, 2006; Dayan, 2005). The higher squalene content in unripe oil (Nsir et al., 2017) provides theoretical basis for enhanced skin benefits.

Limitations and gaps: Evidence for dermatological benefits comes primarily from general olive oil studies, *in vitro* data on individual compounds, or theoretical extrapolation. No clinical trials have assessed topical application of unripe olive oil specifically.

Mechanistic plausibility vs. clinical evidence: While the chemical composition suggests plausibility, this represents theoretical support rather than clinical validation. *In vitro* antimicrobial and antioxidant activities do not necessarily translate to clinical efficacy in complex biological systems.

#### 5.3.3 Antiperspirant claims

Historical claim: Inhibits secretion of sweat from the body.

Modern evidence: Polyphenols demonstrate antibacterial activity against skin microbiota (Nunes et al., 2022), and tannins may act as astringents (Teerasumran et al., 2023).

Critical assessment: The historical claim likely conflates antimicrobial effects with antiperspirant function. Reducing bacterial colonization might diminish odor but does not constitute true perspiration inhibition. The proposed tannin mechanism remains speculative without specific studies on olive oil application for this purpose.

#### 5.3.4 “Astringency” and terminology challenges

Historical sources term perfume base oils “astringents” (στυπτικά) (Pliny XIII.7), yet the sensation attributed to Omphacinon likely refers to pungency from oleocanthal and polyphenolic compounds rather than true mouth-drying astringency familiar from tannin-rich substances. This represents a challenge in interpreting ancient terminology through modern sensory and chemical frameworks. The historical term may describe a complex sensory experience, combining pungency, bitterness, and perceived “sharpness,” that does not map directly onto contemporary astringency as understood in sensory science.

## 5.4 Extraction methods and their effect on oil quality

Ancient sources describe in detail the meticulous production methods of Omphacinon oil. Modern research supports the observation that in manual harvesting, higher levels of polyphenols are preserved compared to mechanical harvesting, and that growing conditions affect the quality of the oil.

Findings from experiments conducted on the traditional “Souri” variety of the Land of Israel show that the level of polyphenols and bitterness decreases with the increase in irrigation doses, and that the highest content was measured in trees that are not irrigated or at low irrigation levels (Bashir, 2009; Dag et al., 2014). These data suggest that the traditional methods of growing and producing Omphacinon oil (rain-fed cultivation, early harvest timing at partial ripeness, manual careful harvesting, and gentle pressing) would have collectively contributed to preserving the high polyphenol content that distinguished this product. The correspondence between traditional practices and modern understanding of factors affecting oil composition indicates empirical optimization based on observable quality characteristics.

The extraction method itself exerts a substantial influence on the phenolic content and overall quality of the resulting oil, and modern process research reveals the significance of what the ancient prescriptions describe in qualitative terms. Malaxation temperature is a critical variable: operating above approximately 40 °C increases oil yield but reduces total polyphenol content and alters the volatile profile, as elevated temperatures accelerate enzymatic degradation of secoiridoids and promote the loss of thermolabile phenolics (Servili et al., 2004). Conversely, short malaxation times at lower temperatures preserve compounds such as oleocanthal and oleuropein. Water addition during malaxation, standard in modern two-phase decanter systems, dilutes the aqueous phenolic fraction and reduces phenolic transfer to the oil; processing conditions that minimise added water and limit oxygen exposure therefore yield oil with higher polyphenol retention. Milling technology also possesses a profound effect on oil quality: while stone-milling produces oils with high phenolic and volatile contents, high-speed hammer mills tend to generate more frictional heat and more extensive cell disruption, which enhance oxidation processes. The temperature of pressing is also significant: cold pressing, now codified in extra-virgin olive oil regulations as below 40 °C, was historically approximated by nocturnal or early-morning harvest and immediate processing, practices described in the Geoponika. Taken together, these findings suggest that the ancient prescriptions for slow, careful pressing of green olives represent an empirically rational approach to maximizing the bioactive content and preserving the quality of Omphacinon, though the underlying chemical mechanisms were not understood in modern terms.

## 5.5 Critical appraisal summary

Strength of evidence for different claims is summarized in Table 1.

The ethnopharmacological value of this traditional knowledge lies primarily in the carrier oil applications and oxidative stability, areas where chemical analysis strongly supports ancient empirical observations. Medicinal claims, while having theoretical basis in olive oil biochemistry, require clinical validation to establish efficacy beyond chemical plausibility.

## 5.6 Health-promoting properties and contemporary preventive medicine

Read together, the chemical data and the historical record agree on several specific points. Polyphenols in unripe olive oil show antimicrobial and antioxidant activity in modern assays, and this fits the oral hygiene uses described by Dioscorides and Pliny; recent meta-analyses of olive oil pulling support the same direction. Squalene, present at higher levels in oil from unripe fruit, is a major component of the human sebum film. This gives a plausible physiological context for the skin uses preserved in rabbinic sources. Taken on these terms, unripe olive oil belongs among the traditional preparations whose health-promoting properties are consistent with current biomedical knowledge and amenable to evidence-based validation through controlled clinical comparison with mature oil. Traditional preparations of this kind are also relevant to Sustainable Development Goal 3 (Good Health and Wellbeing), which encourages the integration of safe, accessible, and culturally embedded health-promoting practices into modern preventive care. Olive oil is widely available, inexpensive, well tolerated, and culturally familiar across the Mediterranean basin and beyond. Whether the unripe form offers measurable advantages for oral and dermatological health is therefore a matter of contemporary public-health interest as well as historical interest. The gap between mechanistic plausibility and clinical demonstration defines the next research step; it does not diminish the value of the traditional record.

## 5.7 Safety considerations and therapeutic limitations

A number of caveats are important. The conclusions reached here identify Omphacinon as a research priority, not as a therapeutic intervention. No controlled clinical trial has been conducted on unripe olive oil for any of the indications recorded in the historical sources, whether oral, dermatological, or gastrointestinal. Dose-response data are equally absent. The ancient sources describe modes of application (rinsing, anointing, ingestion in compound preparations), but the quantities they report do not translate into modern dosing conventions, and the available compositional studies have not been paired with pharmacokinetic work. Tolerability is a further question. Olive oil has a long record of safe dietary and topical use, but the higher polyphenol load of the unripe form, and oleocanthal in particular, can produce a marked pharyngeal irritation; the threshold at which this becomes clinically meaningful in oral or topical use has not been characterized. For these reasons the review does not advise translating any of the historical internal uses into current practice without further safety and efficacy work. The comparative clinical studies proposed here should be understood within standard safety-monitoring frameworks for traditional preparations.

## 6 Conclusion

Omphacinon, the oil obtained from unripe olives, has a documented Mediterranean career stretching across more than a millennium of texts and material remains. The practice was Mediterranean in reach, attested in Greco-Roman medical and agronomic writing and in archaeological remains across the central and eastern Mediterranean; the Levantine sources, while prominent, document its depth rather than define its geography. Its applications are recorded in oral hygiene, in skin care, and in the compound medicinal-aromatic preparations on which Greek, Roman, rabbinic, and Arabic-Islamic medical traditions all relied. Its parallel role as a base for prestige perfumes cannot be cleanly separated from those medical functions, since such perfumes were applied directly to skin and to the oropharyngeal mucosa and were marketed as medicated unguents. What set Omphacinon apart was a particular combination of properties: improved oxidative stability, distinctive sensory character, and unusually long shelf life. Reading those traditional advantages alongside the modern chemistry of the same oil is what this review has tried to do, and the resulting picture supports the rationale behind the use of Omphacinon as a medicinal carrier; it identifies the dimensions in which traditional preference aligns with modern analytical evidence.

The high content of polyphenols, tocopherols, and squalene in unripe olive oil may explain its advantages as a base for perfumes: improved oxidative stability, long shelf life, and the ability to preserve scents. Modern scientific research indicates that unripe olive oil indeed contains higher concentrations of these compounds compared to oil from ripe olives, which gives it enhanced oxidative stability. Relatively low volatile content may explain its suitability as a base for perfumes, allowing intentionally added aromatic compounds to predominate. Unique chemical interactions with various fragrance materials may help explain the preferences for combining it with specific aromatic compounds, though experimental evidence shows these effects are formulation-specific rather than universal.

The antibacterial activity of polyphenolic compounds and the properties of squalene as a skin softener provide a theoretical basis for the diverse cosmetic and dermatological-preventive applications described in ancient sources, though clinical efficacy for specific medicinal uses remains to be demonstrated. Critical appraisal reveals that while carrier oil functionality and oxidative stability advantages find strong chemical support, therapeutic claims require clinical validation beyond mechanistic plausibility.

Historical sources describe meticulous extraction methods, including the selection of fruit at the right stage of partial ripening, careful manual harvesting, and gentle pressing. Archaeological findings, especially evidence for small dedicated pressing facilities discovered at sites such as Pompeii, Delos, and Paestum, support the literary descriptions and indicate the existence of a specialized industry. These facilities would have allowed precise, high-quality extraction of Omphacinon oil suited to the needs of the perfume industry.

Evidence reviewed here suggests that long-standing practical knowledge may, in some cases, reflect real differences in chemical and physical performance, even in the absence of modern analytical frameworks. Correspondence between traditional extraction methods and oil stability, alongside the deliberate pairing of specific base oils with particular fragrance materials, points to a sophisticated empirical practice in ancient perfumery. Where the ethnopharmacological contribution of this review lies, then, is in clarifying which traditional claims find strong chemical support, which remain plausible but undemonstrated, and which are still speculative. More broadly, the case of Omphacinon shows the value of reading historical texts, archaeological evidence, and chemical data together, while maintaining a clear distinction between material plausibility and therapeutic proof.

In light of these findings, future work should examine the effect of unripe olive oil on the stability of volatile fragrance materials in modern perfume systems. Research could focus on quantitative analysis of the interactions between polyphenols from green olives and common aromatic compounds in the perfume industry. The potential of unripe olive oil as a natural component in modern formulations also deserves attention, given the increasing interest in naturally derived ingredients in contemporary cosmetic and personal-care formulations.

From an ethnopharmacological perspective, controlled clinical studies comparing unripe versus mature olive oil for specific traditional applications, particularly oral health and dermatological uses, would be valuable to determine whether the higher polyphenol content translates to enhanced clinical efficacy. Such research should distinguish between chemical composition advantages and actual therapeutic outcomes in human subjects, and would contribute to the broader effort to integrate safe, accessible, traditional preparations into evidence-based preventive care.
