# Supplementary material for: Omphacinon (unripe olive oil) as a traditional Mediterranean medicinal carrier: an ethnopharmacological appraisal
Source: Front Pharmacol. 2026 Aug 5;17:1885776. doi: 10.3389/fphar.2026.1885776 (PMC13485902; doi:10.3389/fphar.2026.1885776)
Supplement: Supplementary file 1 [file DataSheet1.pdf]

Supplementary Material

Preamble

This Supplementary File documents the manuscript's compliance with the Consensus Statement on the Phytochemical Characterisation of Medicinal Plants (ConPhyMP), as defined by Heinrich et al. (2022), Front. Pharmacol. 13:953205, and with the Four Pillars of Best Practice in Ethnopharmacology adopted by Frontiers in Pharmacology for the Ethnopharmacology section.

The manuscript is a narrative ethnopharmacological review and does not present originally generated extraction, analytical, or pharmacological data. Items in the ConPhyMP checklists that pertain to fresh material (voucher specimens, batch numbers, certificates of analysis, originally generated chromatographic data, comparative scoring of in-house samples) are therefore answered "Not applicable", with a brief justification. Items that pertain to taxonomic identification, plant part, mode of historical processing, marker compounds, and reference data are answered substantively, with cross-references to the relevant sections of the manuscript.

Two ConPhyMP tables are provided. Table S1 corresponds to the ConPhyMP recommendations for reporting plant material and its initial processing. Table S2 corresponds to the ConPhyMP analytical-methods recommendations for extracts in international commercial use without a pharmacopoeial monograph specific to the form under study (Type B). The mature olive oil preparation has a European Pharmacopoeia monograph; the unripe-fruit form (Omphacinon) discussed here does not, which makes Type B the appropriate category for the present material.

Four Pillars of Best Practice: brief self-assessment

Pillar 1: Pharmacological context. The review presents the traditional context for Omphacinon based on multiple primary sources across Greek, Latin, Hebrew, Aramaic, and Arabic-Islamic medical traditions. Items relating to dose range, experimental models, and validated assays do not apply, as no original pharmacological experiments are presented; this is stated explicitly in Section 2 (Methodology) and acknowledged in Section 5.7 (Safety Considerations and Therapeutic Limitations).

Pillar 2: Composition. Botanical identification is given as *Olea europaea* L. (Oleaceae), with authentication against MPNS (plant ID 355112; V15, December 2025) and World Flora Online (March 2026) reported in Section 2.3. Chemical composition is summarised in Section 4 and Table 2 with full reference to the cited analytical literature.

Pillar 3: Ethics and contribution. The review draws exclusively on previously published primary historical sources, archaeological reports, and analytical-chemical literature; no new plant material has been collected. Nagoya Protocol and CITES considerations therefore do not apply (item 4a, Table S1). The work brings together textual, archaeological, and analytical evidence around Omphacinon for the first time and identifies a research agenda for comparative clinical work.

Pillar 4: Article-specific requirements (review). The review states a defined research question (Section 1), a search strategy with databases and inclusion criteria (Section 2.3), and a critical appraisal of evidence quality, separating chemical plausibility from clinical demonstration (Sections 5.3, 5.5; Table 3). Limitations and a research agenda are stated in Sections 5.6, 5.7, and 6.

Table S1. ConPhyMP Table 1: Plant material and initial processing

Self-assessment for the manuscript. Not-applicable items are flagged with brief justification.

Item	ConPhyMP recommendation	Response for this manuscript	Manuscript cross-reference
1a	Title	Omphacinon (Unripe Olive Oil) as a Traditional Mediterranean Medicinal Carrier: An Ethnopharmacological Appraisal	Title page
1b	Abstract	Continuous IMRAD paragraph (343 words). Background, methods, results, conclusions, and limitations are reported.	Abstract
2a	Authenticated species name	*Olea europaea* L. (Oleaceae). Verified against the Medicinal Plant Names Services (MPNS), Royal Botanic Gardens, Kew (plant ID 355112; MPNS V15, December 2025) and against World Flora Online (March 2026).	Section 2.3
2b	Pharmacopoeia consistency	Not applicable to the historical preparation under review. Modern olive oil preparations (*Olivae oleum virginale*) have monographs in the European Pharmacopoeia, but the unripe-fruit form (Omphacinon) is not covered by a current pharmacopoeial monograph.	Section 3.1
2c	Locality, date, season of harvesting	Not applicable: review article, no newly generated material. Historical sources describe harvest at partial ripening, approximately three months earlier than the standard ripe-fruit harvest, in Mediterranean olive-growing regions (Columella XII.54; Geoponika IX.19; modern parallel data in Dag et al., 2011).	Section 3.2.1
2d	Voucher specimen	Not applicable: review article, no plant material newly collected, processed, or analysed. Modern reference data are drawn from cited published studies; voucher data are reported in those studies where applicable.	Section 2.3
2e	Authentication method	Not applicable: no new material authenticated. Modern data on unripe olive oil composition are drawn from peer-reviewed sources reporting their own taxonomic and analytical authentication.	Section 2.3; Tables 2, 3
2f	Specific observations on samples	Not applicable. Where ancient sources comment on harvest stage and fruit appearance, these are reported and cited (Columella XII.54; Mishnah	Sections 3.1, 3.2.1

**Item**	**ConPhyMP recommendation**	**Response for this manuscript**	**Manuscript cross-reference**
		Menachot 8:3).	
3a	Full species name with authority and family; international drug name	*Olea europaea* L. [Oleaceae]. International drug name (when used in modern preparations): *Olivae oleum virginale*. The historical material under review (Omphacinon, oil from unripe fruit) does not have a separate international drug name.	Sections 2.3, 3.1
3b	Plant parts used	Drupe (fruit) at unripe / partially ripened stage; pressed without crushing the stones, according to the ancient sources (Columella XII.54; Geoponika IX.19).	Section 3.2
3c	Drug-to-extract ratio (DER) / drug-to-solvent ratio (DSR)	Not applicable: no new extract prepared. Historical yield data cited in the review: 10–15% oil (m/m) from unripe fruit vs. 20–30% from ripe fruit (Dag et al., 2011).	Section 3.2.1
3d	Pre-extraction / fermentation procedures	Not applicable. Historical pre-pressing procedures are described qualitatively (manual harvesting, cleaning, no fermentation).	Section 3.2.2
3e	Extraction solvent	Not applicable. Historical extraction was solvent-free mechanical pressing. Reviewed analytical literature reports its own solvent systems for analytical extractions of the oil.	Section 3.2.2
3f	Type of extract by consistency	Liquid fixed oil (cold-pressed). Not an extract in the modern pharmaceutical sense.	Sections 3.1, 3.2
3g	Mode of extraction	Mechanical cold pressing using a small wedge press (Bodeda) following manual harvest, as described in classical sources and reconstructed archaeologically (Columella XII.54; Geoponika IX.19; Mattingly, 1990; Brun, 2000).	Sections 3.2.2, 3.2.3
3h	Extraction pressure, temperature, duration	Not applicable: review article, no new extraction performed. Historical sources specify gentle pressing at ambient temperature; cited modern studies report their own conditions.	Section 3.2.2
3i	Drying mode	Not applicable. The product is a fixed oil; no drying step.	Section 3.2
3j	Traditional processing	Manual harvesting at partial ripeness; gentle wedge pressing of fruit without crushing the stones; collection of the oil by ladle; storage in a cool, dry place (Columella XII.54; Geoponika IX.19).	Sections 3.2.2, 3.2.3

**Item**	**ConPhyMP recommendation**	**Response for this manuscript**	**Manuscript cross-reference**
3k	Other processing steps	None reported in the historical sources beyond the steps listed under 3j.	Sections 3.2.2, 3.2.3
3l	Ratios in multicomponent preparations	Not applicable to Omphacinon as a single-component oil. Compound perfume and unguent formulations in which Omphacinon serves as a carrier are described qualitatively in the review with reference to the primary sources.	Section 3.3
3m	Standardisation / quantification of marker compounds	Not applicable: no new material standardised. Marker compounds discussed in the review include hydroxytyrosol, oleuropein, tyrosol, oleocanthal, squalene, α-tocopherol, and the C6 LOX-pathway volatiles. Quantification data are taken from the cited primary studies.	Sections 4.1–4.4; Table 2
3n	Content of active ingredients	Not applicable for the historical material. Modern comparative levels (relative trends, unripe vs. mature) are summarised in Table 2 with primary references.	Table 2
3o	Antimicrobial preservatives	None.	:
3p	Excipients	None.	:
4a	Nagoya Protocol / CITES compliance	Not applicable: no new plant material collected. *Olea europaea* L. is not a CITES-listed or threatened species.	Section 2.3
4b	Phytosanitary regulations	Not applicable: no plant material transferred between jurisdictions.	:
4c	Co-authorship from country / region of origin	All co-authors are based in Israel (Bar-Ilan University; Volcani Institute, Agricultural Research Organization), within the Mediterranean region central to the historical sources reviewed.	Author affiliations
5	Commercial product details	Not applicable. The review does not concern a specific commercial product.	:

Table S2. ConPhyMP Table 2b: Analytical methods (Type B)

Self-assessment for the manuscript. The unripe-fruit form (Omphacinon) does not have a pharmacopoeial monograph specific to it, while the mature olive oil preparation does; the appropriate ConPhyMP analytical-methods category for the material under review is therefore Type B.

Item	ConPhyMP recommendation (Type B)	Response for this manuscript	Cross-reference
1	Type of extract	Type B: botanical material in international commercial use without a pharmacopoeial monograph specific to the unripe-fruit form. The mature-fruit oil is covered by a European Pharmacopoeia monograph (Olivae oleum virginale); the unripe-fruit form discussed in this review is not.	Section 2.3
2	Preferred / main methods: triple chemical fingerprinting + quantification of two marker compounds	Not applicable as primary work; review article without new analytical experiments. The cited analytical literature uses the standard methods for unripe olive oil characterisation: HPLC-DAD and HPLC-MS for phenolic profiling (hydroxytyrosol, tyrosol, oleuropein, oleocanthal); GC-MS for volatile profile (C6 LOX-pathway compounds, esters, alcohols, ketones); and saponification followed by GC-FID or HPLC for squalene and tocopherols. Quantification of at least two marker compounds (typically total polyphenols and squalene) is reported in the cited primary studies (Dag et al., 2011; Nsir et al., 2017; Psathas et al., 2022; Karagoz et al., 2017).	Sections 4.1–4.4; Tables 2, 3
3	Alternative methods: single fingerprinting with three detection parameters + quantification of two markers	See item 2. The cited studies typically combine a single chromatographic separation with multiple detectors (DAD + MS or FID + MS), satisfying the alternative-methods recommendation.	Sections 4.1–4.4
4	Use of reference standards	Reported in the cited primary studies. Standards used for the polyphenol fraction commonly include hydroxytyrosol, tyrosol, oleuropein, and oleocanthal; for tocopherols, α- and γ-tocopherol; for the volatile fraction, C6 alcohols and aldehydes.	Section 4.1; Table 2
5	Comparison of different samples / scoring for similarity	Not applicable to the present review. Cited primary studies (e.g. Psathas et al., 2022; Nsir et al., 2017) report comparative chemical fingerprinting between ripening stages within their own datasets; these comparisons provide the basis for the trends	Section 4.2; Table 2

**Item**	**ConPhyMP recommendation (Type B)**	**Response for this manuscript**	**Cross-reference**
		synthesised in Table 2.	

Reference

Heinrich, M., Jalil, B., Abdel-Tawab, M., Echeverria, J., Kulić, Ž., McGaw, L.J., Pezzuto, J.M., Potterat, O., Wang, J.B., 2022. Best practice in the chemical characterisation of extracts used in pharmacological and toxicological research – the ConPhyMP – Guidelines. Front. Pharmacol. 13, 953205.
